# Burnout, Psychological and Musculoskeletal Disorders in Healthcare Assistants Working in Residential Care Homes: A Cross‐Sectional Study in North‐West Italy

**DOI:** 10.1155/jonm/2258099

**Published:** 2026-10-22

**Authors:** Alberto Catalano, Barbara Sodano, Alberto Bentsik, Ilenia Giommarini, Alex Bianchi, Fulvio Ricceri

**Affiliations:** ^1^ Centre for Biostatistics Epidemiology, and Public Health Department of Clinical and Biological Sciences University of Turin Orbassano Turin Italy unito.it; ^2^ Department of Translational Medicine University of Eastern Piedmont Novara Italy uniupo.it; ^3^ Department of Statistics Computer Science, Applications University of Florence Florence Italy unifi.it

**Keywords:** burnout, job insecurity, long-term care workforce, occupational risk factors, psychosocial factors, work-related stress

## Abstract

**Objectives:**

Healthcare assistants (HAs) working in residential care homes are exposed to substantial physical and psychological demands, including heavy workloads and emotionally demanding care tasks. These working conditions may contribute to several adverse health outcomes, such as burnout, work‐related stress, and musculoskeletal pain. This study aimed to investigate the association between socio‐demographic and occupational factors and multiple psychological and physical outcomes among HAs working in residential care homes in North‐West Italy.

**Methods:**

A cross‐sectional study was conducted among 202 female HAs employed in residential care homes in the Piedmont Region. Data were collected through an online, self‐administered, anonymous questionnaire, using validated instruments, including the Job Content Questionnaire, the Patient Health Questionnaire‐2, the Generalized Anxiety Disorder‐7, and the Maslach Burnout Inventory. Multivariable Poisson regression models and multinomial logistic regression models were implemented.

**Results:**

Musculoskeletal pain was highly prevalent, with 86% of participants reporting pain in at least two body sites. Higher levels of musculoskeletal pain were strongly associated with burnout outcomes, particularly emotional exhaustion and depersonalization. Job insecurity was significantly associated with increased risks of depersonalization, emotional exhaustion, and work‐related stress. Working in facilities hosting protected residents was associated with higher prevalence of depression and anxiety. Foreign workers showed lower prevalence of depression and lower risk of depersonalization compared with Italian workers.

**Conclusions:**

These findings highlight the complex relationship between physical strain, organizational factors, and psychological health among HAs. Interventions aimed at reducing biomechanical workload, improving job stability, and strengthening psychological support in residential care settings may contribute to prevent adverse occupational health outcomes.

**Implication for Nursing Management:**

These findings highlight the need for nursing management to implement organizational strategies aimed at reducing physical workload and improving job stability among HAs. Interventions such as adequate staffing, ergonomic support, and structured psychological support services may help prevent burnout and musculoskeletal disorders. Additionally, greater attention should be given to high‐burden care settings, where targeted support and supervision could improve both staff well‐being and quality of care.

## 1. Introduction

Burnout, psychological distress, and musculoskeletal pain are increasingly recognized as major occupational health concerns among healthcare assistants (HAs) [1–3], yet the factors underlying these outcomes remain insufficiently understood, particularly in the Italian context. Support roles in healthcare were introduced into the Italian socio‐healthcare system starting in the 1980s, with their role and training continuously evolving. A measure adopted by the State‐Regions Conference in 2001 established the figure of the HAs (in Italian “Operatore Socio‐Sanitario”, OSS), a professional figure distinct from nurses: while nurses hold a university degree and are responsible for clinical and technical care, HAs complete a regional vocational training pathway and are primarily responsible for assisting residents with activities of daily living, basic hygiene, mobility, and social support, under the supervision of nursing staff [4]. According to the data, to date, approximately 341,000 subjects worked as HAs in Italy, many of whom are employed in around 13,000 residential care homes across the country [5, 6]. Globally, HAs account for 70%–90% of staff in residential care homes and are responsible for up to 90% of the direct care provided to the residents.

The presence of high workload intensity in this sector, also due to the contact with older adults who are often nonautonomous or living with disabilities, appears to be associated with a high prevalence of physical and mental stress resulting from the nature of the work performed [7–9]. In fact, residents in care homes commonly require also assistance in daily activities, such as bathing, dressing, eating, transferring, toileting, and walking [1]. Therefore, working in residential care homes involves not only performing work that is typically organized in shifts, including night shifts and work on public holidays, but also, in many situations, carrying out tasks in settings where staffing levels are inadequate [10]. Additionally, staff may lack the qualifications and preparation required to meet the needs of older residents, a situation that has progressively worsened over the years due to increasingly critical conditions of dependency and health among residents [7]. For the aforementioned reasons, burnout is a relevant issue in HAs.

Maslach conceptualized the burnout syndrome as a three‐dimensional syndrome comprising emotional exhaustion, depersonalization, and reduced personal accomplishment [11]. Emotional exhaustion reflects the depletion of emotional resources, leading individuals to feel that they can no longer invest themselves emotionally in their work. Depersonalization is characterized by cynical or detached attitudes toward patients, whereas reduced personal accomplishment involves a negative self‐assessment of one’s professional competence and effectiveness [11]. According to the literature, HAs appear to be at high risk of burnout, especially younger HAs with fewer years of work experience, whereas good sleep quality, improved unit‐based culture (respect and increased decision‐making with nurses), shorter shifts, and increased social support appear to be the most protective factors against burnout [2]. However, most of the available evidence on burnout among HAs derives from Northern European and North American healthcare systems [12, 13], and comparable studies from Southern Europe, including Italy, remain scarce, despite differences across countries in workforce composition, training pathways, and the organization of long‐term care services that may influence both the prevalence and the determinants of burnout.

Further mental issues that affect HAs are anxiety, depression, and work‐related stress.

A study conducted by Geiger‐Brown et al. showed that more than one in three suffers from depression, and one in four from anxiety. Moreover, depressive and anxiety symptoms were found to be more common among those working 6–7 days per week, among those working two or more double shifts per month, with rotating shifts, and more than 50 h per week [14].

Related to work‐related stress, the literature shows that around 60% of HAs working in residential care homes present symptoms [15–17]. However, very few studies analyzed the main predictors of work‐related stress among HAs.

In addition to psychological issues, many HAs also experience musculoskeletal pain due to the physically demanding nature of their tasks. Some studies observed that most HAs reported musculoskeletal pain involving at least one body part, reaching rates greater than 80% [3, 18, 19].

Although several studies have examined mental or physical health dimensions separately among HAs in residential care homes [2, 3, 8, 9, 18], to our knowledge, no study has simultaneously investigated a broad range of socio‐demographic and occupational predictors in relation to multiple psychological and physical outcomes within the same population, and evidence specific to the Italian context is particularly limited. Clarifying these associations is important both to fill this evidence gap and to inform occupational health policies and prevention strategies tailored to the Italian long‐term care workforce. Therefore, the present study aimed to analyze the association between socio‐demographic and work‐related dimensions and psychological and physical outcomes, including burnout, anxiety, depression, work‐related stress, and musculoskeletal pain, in a population of more than 200 HAs working in residential care homes in North‐West Italy.

## 2. Materials and Methods

### 2.1. Study Population

The study population consisted of HAs who worked in residential care homes in Piedmont Region, North‐Western Italy. Recruitment was carried out with the collaboration of trade unions operating in nursing homes, as well as with the collaboration of nursing home management. A presentation of the study was made to all regional trade unions, and a letter describing the project was sent to all employers. When availability was given, recruitment took place during union meetings, after a brief presentation of the research being conducted. Data were collected through an online, self‐administered questionnaire, administered using LimeSurvey (https://www.limesurvey.org). Participation was voluntary and fully anonymous: the platform was configured not to collect any personal identifying information, in compliance with the General Data Protection Regulation (GDPR), and questionnaires could not be linked to individual respondents.

### 2.2. Outcome of Interest

In this study, nine different outcomes were analyzed.

First, we investigated musculoskeletal pain, defined as a composite variable derived from 11 different items: shoulder pain, elbow pain, wrist/hand pain, cervical pain, dorsal pain, low back pain, knee pain, foot pain, daily paresthesia, nocturnal paresthesia, and acute low back pain. This variable ranged between 0 and 11, corresponding to the number of sites affected by musculoskeletal pain, and it was categorized into three different levels: 0–1, 2–4, and 5+.

Then, we investigated work‐related stress (job strain), assessed using the short (14‐item) version of the Job Content Questionnaire (JCQ) [20], comprising the Psychological Job Demands (5 items) and Decision Latitude (9 items, combining Skill Discretion and Decision Authority) subscales. Job strain was computed from these two subscales, and participants were classified as exposed to work‐related stress if their score fell above the median value observed in the present study sample. This approach was preferred over the classic quadrant‐based classification because no validated normative cut‐offs for the JCQ subscales are available for this specific occupational population in Italy, and applying externally derived reference could have led to misclassification of job strain in our sample. We also measured depression and anxiety. Depression was measured through the Patient Health Questionnaire‐2 (PHQ‐2) [21], and scores of 3+ indicated the presence of depressive symptoms and anxiety was evaluated through the Generalized Anxiety Disorder‐7 (GAD‐7) [22]. A score of 10+ reflected the presence of anxiety symptoms.

Lastly, we investigated burnout, assessed using the 22‐item Maslach Burnout Inventory (MBI), using the validated Italian version by Sirigatti and Stefanile [23]. We evaluated the three different components of burnout both separately and combined. Emotional exhaustion was measured using the nine items associated with this dimension and stratified into three categories: ≤ 13 (low), 14–22 (moderate), and ≥ 23 (high). Depersonalization was measured using five MBI items and categorized into three levels: ≤ 2 (low), 3–5 (moderate), and ≥ 6 (high). For both dimensions, lower scores corresponded to lower levels of emotional exhaustion and depersonalization, respectively. Personal accomplishment was assessed using the remaining eight items and categorized as high (≥ 39), moderate [24–29], and low (≤ 31) level of personal accomplishment. Finally, we defined the risk of burnout as having the highest level in at least two of the three dimensions, whereas severe burnout was defined as having the highest level in all three dimensions.

### 2.3. Measure of the Exposures

Regarding the potential predictors for these outcomes, they included: nationality (Italian, foreign workers from EU countries and foreign workers from non‐EU countries), age, smoking habits, physical activity (expressed as number of hours per week), BMI, calculated as weight in kilograms divided by height in meters squared and categorized according to WHO cut‐offs as underweight/normal weight (< 25.0 kg/m^2^), overweight (25.0–29.9 kg/m^2^), or obese (≥ 30.0 kg/m^2^), with underweight participants combined with the normal‐weight category due to their small number, social support, that was measured through the Copenhagen Psychosocial Questionnaire (COPSOQ) [30] and dichotomized by the median, working in a residential care facility hosting independent residents, residents with addictions, protected residents, or residents with disabilities, job insecurity related to employment stability, and presence of financial difficulties. In addition to these factors, work‐related stress was included as a predictor in the analyses of all other outcomes investigated, while musculoskeletal pain was included when analyzing psychological and burnout outcomes.

### 2.4. Statistical Analyses

All variables of interest were described using median and interquartile range (IQR) or frequencies and percentages, based on the nature of the variables (quantitative or qualitative, respectively).

To investigate the association between each predictor and outcome, for the analysis which involved binary endpoints, multivariable Poisson regression models were fitted, by estimating prevalence ratios (PRs) and related 95% confidence intervals (CIs). This approach was preferred over log‐binomial regression, which, although also suited to directly estimate risk ratios, is more prone to convergence failure, which can occur in models with several categorical predictors and a moderate sample size, as in the present study. Poisson regression with robust variance estimation is a well‐established and widely used alternative that avoids these convergence limitations while providing valid point estimates and CIs for the PR [31]. In the case of nonbinary outcomes, instead, multivariable multinomial logistic regression models were performed to estimate relative risk ratios (RRRs) and related 95% CIs. Associations were considered statistically significant when the 95% CI did not include 1. Estimates that could not be computed reliably because of sparse data (zero or near‐zero cell counts) are reported as not estimable (NE).

As a sensitivity analysis, age was additionally modeled as a categorical variable with three groups of approximately equal size (≤ 44, 45–54 [reference], and ≥ 55 years, corresponding to tertiles of the observed age distribution) to explore potential nonlinear associations with the outcomes investigated.

All analyses were conducted using STATA (V.19) and *R* (V.4.2).

## 3. Results

The study included a total of 202 women HAs working in nursing homes. The median age of participants was 50 years (IQR: 41–56). Most women were of Italian nationality (60%), while 17% were from other European Union countries and 23% from non‐EU countries. Musculoskeletal pain was highly prevalent in the sample: 48% of participants reported pain in 2–4 body sites and 39% reported pain in 5 or more sites. Almost half of the women experienced work‐related stress (44%), and more than half were classified as being at risk of burnout (51%). High levels of burnout dimensions were common, particularly emotional exhaustion (73%) and depersonalization (58%), while severe burnout was identified in 7% of participants. Symptoms of depression and anxiety were reported by 21% and 24% of the sample, respectively. Regarding lifestyle and occupational characteristics, 31% of women were current smokers, 60% reported no regular physical activity, and 56% were overweight or obese. Most participants worked in nursing homes with protected units (83%) and units for residents with physical disabilities (58%), while job insecurity was reported by approximately one‐third of the sample (33%) (Table 1).

**TABLE 1:** Characteristics of the study participants (*n* = 202).

Characteristic	*n* (%)
*Socio-demographic and lifestyle characteristics*	
Age, years, median (IQR)	50 (41–56)
Nationality	
Italian	121 (60)
EU countries	35 (17)
Non‐EU countries	46 (23)
Financial difficulties	165 (82)
Current smoker	63 (31)
Physical activity	
None	121 (60)
1–2 h/week	55 (27)
≥ 3 h/week	26 (13)
Body mass index	
Normal weight	89 (44)
Overweight	78 (39)
Obese	35 (17)
*Work-related characteristics*	
Nursing home with units for	
Independent residents	56 (28)
Residents with physical disabilities	117 (58)
Protected residents	167 (83)
Residents with addictions	11 (5)
Job insecurity	66 (33)
High social support	131 (65)
*Health outcomes*	
Musculoskeletal pain, number of sites	
0–1	28 (14)
2–4	96 (48)
≥ 5	78 (39)
Work‐related stress	89 (44)
Depression	42 (21)
Anxiety	49 (24)
Emotional exhaustion	
Low (≤ 13)	27 (13)
Moderate (14–22)	27 (13)
High (≥ 23)	148 (73)
Depersonalization	
Low (≤ 2)	60 (30)
Moderate (3–5)	25 (12)
High (≥ 6)	117 (58)
Personal accomplishment	
Low (≤ 31)	134 (66)
Moderate (32–38)	49 (24)
High (≥ 39)	19 (9)
Burnout risk	103 (51)
Severe burnout	14 (7)

*Note:* Values are numbers (percentages) unless otherwise indicated. Percentages may not sum to 100 because of rounding. Nursing home unit types are not mutually exclusive. Body mass index: normal weight < 25.0 kg/m^2^ (including underweight), overweight 25.0–29.9 kg/m^2^, obese ≥ 30.0 kg/m^2^. High social support: score above the sample median (Copenhagen Psychosocial Questionnaire). Musculoskeletal pain: number of body sites with pain (range 0–11). Work‐related stress: job strain score above the sample median (> 37; Job Content Questionnaire). Depression: Patient Health Questionnaire‐2 score ≥ 3. Anxiety: Generalized Anxiety Disorder‐7 score ≥ 10. Emotional exhaustion, depersonalization, and personal accomplishment were assessed with the Maslach Burnout Inventory (score ranges in brackets). Burnout risk: high emotional exhaustion, high depersonalization, or low personal accomplishment in at least two of the three dimensions; severe burnout: in all three dimensions. IQR, interquartile range.

Abbreviation: EU, European Union.

The results related to the association between each predictor and outcome are summarized in Table 2, Table 3, and Supporting Table S1. In multivariable analyses, several statistically significant associations emerged between health‐related, occupational, and organizational factors and both burnout‐related outcomes and musculoskeletal pain. Musculoskeletal pain was strongly associated with burnout dimensions. Compared with women reporting 0–1 musculoskeletal painful site, those reporting a higher number of painful sites showed consistently worse burnout profiles. Women reporting 5 or more musculoskeletal pain sites exhibited a significantly increased relative risk of high depersonalization (RRR = 3.21, 95% CI: 1.03–9.97) and high emotional exhaustion (RRR = 4.29, 95% CI: 1.29–14.28). In line with these findings, higher levels of musculoskeletal pain were also associated with an increased prevalence of overall burnout risk, with a PR of 1.92 (95% CI: 1.07–3.43) for 5 or more painful sites, compared with the reference category.

**TABLE 2:** Association of participant characteristics with binary outcomes: prevalence ratios (95% CI) from multivariable Poisson regression.

Characteristic	Work‐related stress	Depression	Anxiety	Burnout risk	Severe burnout
*Socio-demographic and lifestyle characteristics*					
Age (per year)	0.99 (0.97–1.00)	1.01 (0.98–1.03)	1.01 (0.99–1.04)	0.99 (0.98–1.01)	1.02 (0.97–1.07)
Nationality					
Italian	Ref	Ref	Ref	Ref	Ref
EU countries	1.02 (0.67–1.54)	0.72 (0.32–1.63)	0.80 (0.38–1.68)	1.10 (0.77–1.56)	0.35 (0.06–2.16)
Non‐EU countries	0.94 (0.61–1.45)	0.35 (0.14–0.87)	0.49 (0.21–1.11)	0.65 (0.42–1.01)	0.26 (0.04–1.76)
Financial difficulties	0.80 (0.55–1.17)	0.88 (0.44–1.77)	1.69 (0.78–3.67)	1.02 (0.70–1.49)	1.28 (0.19–8.41)
Current smoker	1.06 (0.74–1.51)	1.29 (0.74–2.25)	1.47 (0.88–2.46)	1.10 (0.82–1.47)	0.74 (0.26–2.13)
Physical activity					
None	Ref	Ref	Ref	Ref	Ref
1–2 h/week	1.18 (0.82–1.68)	1.07 (0.56–2.08)	0.70 (0.36–1.34)	1.00 (0.72–1.39)	2.29 (0.63–8.25)
≥ 3 h/week	1.08 (0.68–1.70)	2.10 (1.16–3.79)	1.12 (0.64–1.97)	1.05 (0.73–1.51)	2.66 (0.79–8.93)
Body mass index					
Normal weight	Ref	Ref	Ref	Ref	Ref
Overweight	1.11 (0.79–1.54)	1.26 (0.70–2.28)	1.12 (0.64–1.94)	1.14 (0.84–1.55)	0.90 (0.22–3.62)
Obese	0.87 (0.53–1.42)	0.90 (0.45–1.81)	0.83 (0.42–1.62)	1.40 (1.02–1.90)	1.69 (0.39–7.38)
*Work-related characteristics*					
Nursing home with units for					
Independent residents	1.04 (0.73–1.49)	0.74 (0.39–1.42)	0.77 (0.43–1.38)	0.70 (0.48–1.01)	1.37 (0.37–5.08)
Residents with physical disabilities	1.10 (0.78–1.56)	1.67 (0.97–2.88)	1.66 (0.98–2.81)	1.10 (0.81–1.50)	0.73 (0.26–2.08)
Protected residents	0.98 (0.65–1.48)	5.27 (1.40–19.86)	2.65 (1.15–6.12)	0.84 (0.61–1.16)	0.44 (0.14–1.37)
Residents with addictions	1.51 (0.87–2.61)	0.72 (0.20–2.56)	0.60 (0.19–1.93)	1.61 (1.06–2.46)	NE
Job insecurity	1.46 (1.05–2.01)	1.54 (0.91–2.62)	1.52 (0.91–2.54)	1.32 (1.01–1.74)	1.63 (0.60–4.42)
High social support	1.05 (0.75–1.46)	0.86 (0.50–1.47)	0.69 (0.42–1.12)	0.87 (0.66–1.14)	1.48 (0.56–3.90)
*Physical and psychological health*					
Musculoskeletal pain, number of sites					
0–1	Ref	Ref	Ref	Ref	Ref
2–4	1.21 (0.71–2.07)	1.91 (0.67–5.39)	1.62 (0.62–4.18)	1.75 (0.99–3.11)	2.84 (0.45–17.74)
≥ 5	1.34 (0.78–2.29)	1.63 (0.54–4.87)	1.95 (0.73–5.20)	1.92 (1.07–3.43)	1.89 (0.28–12.57)
Work‐related stress	—	1.44 (0.83–2.49)	2.19 (1.34–3.57)	1.26 (0.97–1.63)	0.85 (0.29–2.48)

*Note:* Values are prevalence ratios (PR) with 95% confidence intervals (CI), estimated with Poisson regression with robust variance. Each column is a separate model including all characteristics listed in the table (work‐related stress was not included in the model for work‐related stress). For binary characteristics, the reference category is the absence of the characteristic (for high social support: social support at or below the sample median). Outcome definitions are given in Table 1. Ref, reference category; NE, not estimable because of sparse data; –, not applicable.

Abbreviation: EU, European Union.

**TABLE 3:** Association of participant characteristics with burnout dimensions: relative risk ratios (95% CI) from multivariable multinomial logistic regression.

Characteristic	Depersonalization		Emotional exhaustion		Personal accomplishment	
	Moderate	High	Moderate	High	Moderate	Low
*Socio-demographic and lifestyle characteristics*						
Age (per year)	1.05 (0.99–1.11)	0.99 (0.96–1.02)	0.97 (0.91–1.03)	0.97 (0.93–1.02)	0.98 (0.94–1.02)	0.99 (0.94–1.06)
Nationality						
Italian	Ref	Ref	Ref	Ref	Ref	Ref
EU countries	1.24 (0.28–5.54)	1.85 (0.64–5.38)	1.17 (0.21–6.44)	1.04 (0.29–3.71)	0.13 (0.03–0.61)	0.37 (0.08–1.62)
Non‐EU countries	0.94 (0.27–3.23)	0.39 (0.16–0.98)	1.11 (0.23–5.40)	0.49 (0.12–1.94)	0.69 (0.28–1.68)	0.28 (0.05–1.67)
Financial difficulties	0.35 (0.10–1.24)	0.62 (0.22–1.74)	0.94 (0.21–4.24)	1.33 (0.44–4.02)	0.62 (0.24–1.59)	0.66 (0.15–2.88)
Current smoker	1.33 (0.38–4.73)	1.38 (0.60–3.16)	1.80 (0.44–7.35)	1.68 (0.54–5.21)	0.82 (0.39–1.72)	0.77 (0.24–2.40)
Physical activity						
None	Ref	Ref	Ref	Ref	Ref	Ref
1–2 h/week	0.95 (0.29–3.17)	0.94 (0.42–2.12)	0.75 (0.17–3.28)	1.02 (0.32–3.23)	0.83 (0.35–1.95)	2.44 (0.70–8.54)
≥ 3 h/week	3.76 (0.89–15.87)	1.78 (0.53–5.94)	0.85 (0.14–5.04)	0.46 (0.09–2.29)	0.45 (0.12–1.75)	3.05 (0.84–11.11)
Body mass index						
Normal weight	Ref	Ref	Ref	Ref	Ref	Ref
Overweight	2.78 (0.82–9.39)	2.75 (1.25–6.03)	2.33 (0.50–10.86)	1.09 (0.34–3.50)	0.92 (0.40–2.12)	0.74 (0.18–3.05)
Obese	0.47 (0.07–3.02)	2.02 (0.77–5.31)	0.66 (0.11–3.84)	0.32 (0.09–1.14)	2.12 (0.75–5.99)	3.52 (0.82–15.08)
*Work-related characteristics*						
Nursing home with units for						
Independent residents	0.81 (0.24–2.71)	0.42 (0.17–1.04)	0.40 (0.08–2.01)	0.13 (0.03–0.53)	1.22 (0.50–3.01)	1.67 (0.47–5.98)
Residents with physical disabilities	1.08 (0.34–3.48)	1.21 (0.55–2.67)	1.70 (0.43–6.63)	1.77 (0.54–5.83)	0.84 (0.36–1.95)	0.40 (0.12–1.34)
Protected residents	2.80 (0.65–11.97)	0.80 (0.31–2.06)	5.12 (0.46–56.84)	0.99 (0.30–3.33)	1.42 (0.49–4.07)	0.36 (0.10–1.35)
Residents with addictions	11.85 (0.33–424.71)	12.26 (0.57–264.89)	NE	7.77 (0.81–74.15)	0.42 (0.06–2.73)	NE
Job insecurity	6.10 (1.98–18.76)	3.47 (1.41–8.53)	4.84 (0.75–31.23)	9.04 (2.05–39.94)	1.30 (0.56–3.03)	1.44 (0.51–4.07)
High social support	1.84 (0.51–6.65)	1.04 (0.47–2.29)	0.87 (0.17–4.36)	0.36 (0.09–1.44)	1.13 (0.49–2.61)	1.74 (0.66–4.60)
*Physical and psychological health*						
Musculoskeletal pain, number of sites						
0–1	Ref	Ref	Ref	Ref	Ref	Ref
2–4	0.71 (0.16–3.12)	2.92 (0.99–8.63)	10.42 (1.39–77.88)	5.09 (1.36–19.10)	1.70 (0.53–5.46)	2.40 (0.44–13.08)
≥ 5	0.76 (0.20–2.90)	3.21 (1.03–9.97)	4.14 (0.63–27.18)	4.29 (1.29–14.28)	3.04 (0.92–10.07)	2.23 (0.36–13.89)
Work‐related stress	0.74 (0.26–1.65)	1.25 (0.61–2.59)	0.88 (0.24–3.21)	1.36 (0.49–3.76)	0.77 (0.36–1.65)	0.73 (0.25–2.09)

*Note:* Values are relative risk ratios (RRR) with 95% confidence intervals (CI). Each burnout dimension was analyzed in a separate model including all characteristics listed in the table. Reference outcome categories: low depersonalization (≤ 2), low emotional exhaustion (≤ 13), and high personal accomplishment (≥ 39). Moderate and high depersonalization: 3–5 and ≥ 6; moderate and high emotional exhaustion: 14–22 and ≥ 23; moderate and low personal accomplishment: 32–38 and ≤ 31. For binary characteristics, the reference category is the absence of the characteristic (for high social support: social support at or below the sample median). Ref, reference category; NE, not estimable because of sparse data.

Abbreviation: EU, European Union.

Job insecurity represented one of the strongest occupational determinants of adverse mental health outcomes. Women reporting insecure employment conditions had higher risks of depersonalization, both at moderate (RRR = 6.10, 95% CI: 1.98–18.76) and high levels (RRR = 3.47, 95% CI: 1.41–8.53), as well as substantially higher risks of emotional exhaustion, particularly at the highest severity level (RRR = 9.04, 95% CI: 2.05–39.94). Moreover, job insecurity was associated with a higher prevalence of work‐related stress (PR = 1.46, 95% CI: 1.05–2.01) and an increased prevalence of burnout risk overall (PR = 1.32, 95% CI: 1.01–1.74).

With respect to body mass index, obesity showed a mixed pattern of associations. Obese women had a significantly lower relative risk of reporting moderate levels of musculoskeletal pain (RRR = 0.26, 95% CI: 0.07–0.90) compared with normal‐weight women; however, obesity was simultaneously associated with a higher prevalence of burnout risk (PR = 1.40, 95% CI: 1.02–1.90). Regarding organizational characteristics, the presence of protected units within the nursing home was strongly associated with poorer mental health outcomes, including a markedly increased prevalence of depression (PR = 5.27, 95% CI: 1.40–19.86) and anxiety (PR = 2.65, 95% CI: 1.15–6.12). Additionally, work‐related stress was itself significantly associated with anxiety, with stressed women exhibiting more than double the prevalence of anxiety compared with those without work‐related stress (PR = 2.19, 95% CI: 1.34–3.57).

Finally, nationality was associated with several outcomes. Compared with Italian women, non‐EU foreign workers showed a lower prevalence of depression (PR = 0.35, 95% CI: 0.14–0.87) and a reduced relative risk of high depersonalization (RRR = 0.39, 95% CI: 0.16–0.98). In contrast, women from EU countries demonstrated higher levels of personal accomplishment compared with Italian workers, with a significantly lower relative risk of moderate reductions in personal accomplishment (RRR = 0.13, 95% CI: 0.03–0.61). No statistically significant associations were observed for age, smoking status, physical activity, financial difficulties, or social support across most outcomes.

In a sensitivity analysis modeling age in three categories, women aged ≤ 44 years showed a significantly higher prevalence of work‐related stress compared with those aged 45–54 years (PR = 1.6, 95% CI: 1.1–2.4), and women aged ≥ 55 years showed a significantly higher relative risk of high emotional exhaustion compared with those aged 45–54 years (RRR = 5.2, 95% CI: 1.1–24.6); no other statistically significant differences by age category emerged across the remaining outcomes.

## 4. Discussion

This study investigated the association between different socio‐demographic and work‐related potential predictors and psychological and physical outcomes in a population involving 202 women HAs operating in residential care homes located in North‐West Italy. Overall, musculoskeletal pain was strongly associated with burnout; job insecurity emerged as one of the strongest occupational determinants of adverse mental health outcomes; a higher BMI was associated with a higher risk of burnout; working in facilities hosting protected residents was associated with higher levels of depression and anxiety; and foreign‐born workers generally showed lower levels of depersonalization and depression, and higher personal accomplishment, compared with Italian workers. A higher number of body sites presenting pain was consistently associated with burnout, in particular with the risk of burnout and with emotional exhaustion. This is consistent with previous evidence linking musculoskeletal complaints and burnout among nursing personnel [32, 33]. In a study conducted among Norwegian workers across several occupational groups, including nurses, the exhaustion dimension of burnout was significantly and positively associated with musculoskeletal pain regardless of profession or gender; among nurses specifically, heavy lifting was identified as a likely contributor to musculoskeletal pain, and emotionally demanding work tasks were hypothesized to be the main contributing factor to burnout in this occupational group [33].

A further possible mechanism linking these two outcomes is that burnout symptoms may result from work‐related accidents, such as sprains, fractures, and contusions, which are themselves strongly associated with musculoskeletal pain [32].

An alternative, non‐mutually exclusive explanation involves the possibility of reverse causality, whereby burnout itself may increase the risk of musculoskeletal pain rather than resulting from it. In a cohort study of healthy employed adults, burnout increased the risk of developing musculoskeletal pain at multiple sites over time [34]. Although the underlying pathophysiological mechanisms have not been specifically investigated, potential pathways may include the effect of high mental workload, which can increase muscle tension and fatigue even under low physical loads [35], and the possibility that job stress may hinder recovery by preventing muscles from relaxing during breaks and after work [34]. Job insecurity was associated with an increased risk of burnout, particularly the depersonalization and emotional exhaustion dimensions, and with a higher prevalence of work‐related stress. Moreover, a similar relationship was found when investigating the association between job insecurity and work‐related stress. This association may be due to the fact that individuals without a permanent contract may experience insecurity arising from uncertainty about their future employment prospects, which may influence their levels of stress and emotionality. However, this association remains debated in the literature: a systematic review reported that workers with a temporary contract and greater worries about job security had lower mean burnout scores [2]. It has been suggested that permanent employees may instead be more vulnerable to routine, monotony, and reduced task variety over time, which could contribute to decreased job satisfaction and increased vulnerability to work‐related stress or burnout [36], although this remains a plausible but untested interpretation in our data, as routine and task variety were not directly measured in this study. A similar relationship was found when investigating the association between job security and work‐related stress, even if slightly higher in magnitude [36]. Although we did not observe a statistically significant association between financial difficulties and burnout in our sample, one previous study among community nurses reported that workers experiencing economic difficulties were more likely to feel exhausted at work [37]; this discrepancy may reflect limited statistical power in our sample and warrants further investigation in larger studies.

Regarding the relationship between BMI and burnout, our findings demonstrated that a higher BMI is associated with higher risk of burnout, especially considering the depersonalization domain. In the literature, strong associations between BMI and different burnout dimensions were found, even if the majority of studies were conducted using a cross‐sectional design, making it difficult to understand the real direction of the effect [24, 25]. One possible explanation is that overweight or obese workers may experience greater physical fatigue in physically demanding roles such as nursing and care work, which could in turn reduce enthusiasm and increase vulnerability to burnout [24]; this interpretation, however, remains speculative in the absence of direct measures of fatigue in our study. Conversely, a reverse relationship is also plausible: individuals experiencing burnout may be more susceptible to emotional or uncontrolled eating behaviors and may show a reduced capacity to modify their eating patterns, which could contribute to weight gain over time [25].

An inverse relationship was observed between BMI and musculoskeletal pain, even if a clear trend is not present. This result contrasts with one previous study reporting that overweight or obese workers are more likely to experience musculoskeletal pain and related symptoms [26], possibly reflecting the greater mechanical load that individuals with higher BMI may experience during physically demanding nursing or care‐home tasks. Given the inconsistency between our findings and this expected mechanism, this result should be interpreted with caution and warrants confirmation in larger samples. When examining the impact of the presence of protected patients in the unit where HAs are employed (e.g., Alzheimer’s disease or dementia), a significant positive association was found with depression and anxiety scales. In another study conducted on this topic, it was observed that more than 50% of caregivers have shown high anxiety and depression scores [27]. The burden of responsibility significantly increases in relation to the complexity of the diseases that affect the patients, leading to greater levels of anxiety and depression [28]. Moreover, when considering the presence of addicted patients in the care homes, we identified a positive relationship with risk of burnout. Similarly, the workload of care‐dependent patients may lead to greater scores of burnout. Workload and emotional burden may also contribute to the association that we observed between work‐related stress and anxiety, whereas the association with depression was in the same direction but did not reach statistical significance (Table 2). These findings are consistent with those reported among Italian radiologists, in whom higher levels of work‐related stress were associated with a greater likelihood of both anxiety and depressive symptoms [29]. Although radiologists and HAs differ markedly in training, tasks, and working conditions, the consistency of these findings suggests that the link between work‐related stress and common mental health symptoms extends across healthcare occupations and supports the inclusion of work‐related stress assessment in mental health prevention programs for HAs.

Finally, our research focused also on the relationship between HAs’ nationality and psychological and physical domains. Results showed that foreign workers had a higher level of personal satisfaction compared with Italian HAs, and lower scores of depersonalizations and depression. These findings may be interpreted considering the so‐called healthy migrant effect. This framework suggests that individuals who migrate are often positively self‐selected in terms of physical and psychological resources, including resilience, motivation, and coping abilities. As a result, migrant workers may initially display better mental health outcomes compared to the native population [38]. Moreover, in some cultural contexts, depressive symptoms may be less openly expressed, as psychological distress carries greater stigma. Individuals may also be more likely to somaticize their emotional difficulties, reporting physical rather than emotional complaints. These factors could contribute to lower self‐reported levels of depression and depersonalization among foreign workers, even if underlying psychological issues are present [39]. Moreover, research indicates that migrant populations may exhibit higher levels of resilience when facing adversity, potentially due to prior exposure to challenging life circumstances [40–42].

Because age showed no association with the outcomes investigated when modeled as a continuous variable, we additionally tested it in three categories (≤ 44, 45–54, and ≥ 55 years) to explore potential nonlinear or U‐shaped effects. This exploratory analysis suggested a possible U‐shaped pattern: younger workers (≤ 44 years) showed a higher prevalence of work‐related stress, while older workers (≥ 55 years) showed a higher risk of high emotional exhaustion, relative to the middle‐aged group (45–54 years). However, these findings should be interpreted with caution, as the CIs were wide, reflecting the relatively small size of the age subgroups. Moreover, job strain was computed from two subscales, the Psychological Job Demands (5 items) and the Decision Latitude (9 items, combining Skill Discretion and Decision Authority), and participants were classified as exposed to work‐related stress if their score fell above the median value observed in the present study sample. This approach differs from the classic quadrant‐based classification of job strain, which typically uses median splits derived from a reference occupational population; this methodological difference, together with the specific occupational demands of this workforce, may explain the higher prevalence of work‐related stress observed in our sample compared with the approximately 25% commonly reported when a classic quadrant approach is applied.

This research has some limitations and strengths.

Its cross‐sectional design may lead to the possibility of reverse causality, and this makes it challenging to draw conclusions about causal associations. Moreover, some information that are potentially relevant predictors, such as distance from home and family, or some lifestyle indicators, were not collected. The relatively modest sample size (*n* = 202), and the small number of participants in some subgroups (e.g., severe burnout, affecting only 14 women), limited the precision of several estimates, as reflected in the wide CIs observed for some associations, and reduced statistical power to detect associations with less prevalent outcomes. Nevertheless, despite this modest sample size, this is one of the first studies conducted in Italy to focus exclusively on HAs working in residential care homes, examining multiple physical and psychological outcomes and a broad range of predictors within the same population. Additionally, although full anonymity was ensured during recruitment, it is possible that women who felt more vulnerable or concerned about potential repercussions may have been less likely to participate. Finally, all participants in our sample were women. This is consistent with the strong female predominance of the long‐term care workforce in Europe, where approximately four‐fifths of employees are female [7], and reflects the composition of the workforce at the participating facilities rather than a sex‐based inclusion or exclusion criterion, although it limits our ability to examine potential sex‐based differences in the associations studied and constrains the generalizability of our findings to male HAs.

## 5. Implications for Practice and Future Research

These findings suggest the potential value of targeted interventions in residential care homes to protect and promote the physical and mental well‐being of HAs, although the cross‐sectional design of this study means these should be regarded as hypotheses for future research and practice rather than as directly demonstrated effects. Organizational measures aimed at preventing musculoskeletal disorders, such as ergonomic improvements, systematic risk assessments, and adequate availability of lifting devices, may be worth evaluating, alongside realistic workload planning that allows sufficient time for task completion and recovery, which could help reduce cumulative biomechanical overload and chronic pain.

Programs to monitor and reduce work‐related stress and burnout could also be evaluated in future research; these might include early identification of distress symptoms and confidential psychological‐support pathways, which may encourage HAs to seek help without fear of stigma or professional repercussions, although the effectiveness of such interventions was not tested in this study. Particular attention may be needed for units caring for highly dependent or protected residents, where both emotional burden and workload appear elevated, additional staffing support and structured team supervision could plausibly reduce emotional strain and support a positive team climate, though these strategies should be formally evaluated rather than assumed to be effective.

Future longitudinal studies are needed to clarify the direction of the associations observed here and to better understand the temporal relationship between physical strain, organizational factors, and psychological outcomes. Intervention studies evaluating the effectiveness of ergonomic, organizational, and psychological prevention strategies would provide valuable evidence to guide occupational health policies and workplace practices in residential care settings.

## 6. Conclusions

In this cross‐sectional study conducted among 202 women HAs working in residential care homes in North‐West Italy, both physical and psychological distress emerged. Musculoskeletal pain was found to be correlated with adverse psychological outcomes, in particular emotional exhaustion and burnout risk. Job insecurity and work organization factors of the residential care homes, such as working in facilities with protected units, were associated with poorer psychological outcomes, including emotional exhaustion, depersonalization, anxiety, depression, and work‐related stress. In addition, higher BMI was associated with a higher risk of burnout. Regarding the nationality of the HAs, foreign workers showed lower levels of depression and depersonalization, probably reflecting selection mechanisms and cultural differences in symptom reporting. However, caution is required in interpreting these results, as under‐reporting due to stigma or different cultural expressions of distress cannot be excluded.

Even if the cross‐sectional design of the study precludes the possibility to establish a causal relationship, our findings underline the existence of a clear relationship between different predictors and physical and psychological outcomes among HAs, which merit attention in future research and practice.

By simultaneously examining multiple physical and psychological outcomes, it offers a broader understanding of the complex health challenges affecting this professional group and provides a foundation for the development of prevention programs aimed at improving both workers’ wellbeing and quality of care.

## Funding

The project was funded by the Ministry of University, PRIN PNRR project “InMigrHealth: Investigating Migrants’ Occupational Health” (P20225JPYN).

## Disclosure

Fulvio Ricceri is the last author.

## Ethics Statement

The study was conducted in accordance with the Declaration of Helsinki, and it was approved by the Ethical Committee of the University of Turin.

## Conflicts of Interest

The authors declare no conflicts of interest.

## Supporting Information

Additional supporting information can be found online in the Supporting Information section.

## Supporting information


**Supporting Information** Table S1. Association of socio‐demographic, lifestyle, and work‐related characteristics with musculoskeletal pain (2–4 and ≥ 5 painful body sites vs 0–1) among healthcare assistants working in residential care homes. Relative risk ratios (RRR) and 95% confidence intervals are estimated from a multivariable multinomial logistic regression model.

## Data Availability

Raw data cannot be openly shared. However, researchers can obtain aggregated data by reaching out to the corresponding author upon request.
