# Supplementary material for: Burnout, Psychological and Musculoskeletal Disorders in Healthcare Assistants Working in Residential Care Homes: A Cross‐Sectional Study in North‐West Italy
Source: J Nurs Manag. 2026 Oct 22;2026:2258099. doi: 10.1155/jonm/2258099 (PMC13691291; doi:10.1155/jonm/2258099)
Supplement: Supplementary file 1 [file JONM-2026-2258099-s001.docx]

**Supplementary Material**

**Table S1.** Association of participant characteristics with musculoskeletal pain: relative risk ratios (95% CI) from multivariable multinomial logistic regression

| **Characteristic** | | **Number of painful sites (vs 0–1)** | |
| --- | --- | --- | --- |
|  | | **2–4** | **≥5** |
| *Socio-demographic and lifestyle characteristics* | | | |
| Age (per year) | | 0.99 (0.95–1.04) | 1.03 (0.99–1.08) |
| Nationality | |  |  |
|  | Italian | Ref | Ref |
|  | EU countries | 1.98 (0.51–7.66) | 0.67 (0.15–2.99) |
|  | Non-EU countries | 1.90 (0.62–5.82) | 0.80 (0.25–2.54) |
| Financial difficulties | | 1.65 (0.56–4.90) | 1.65 (0.50–5.47) |
| Current smoker | | 2.50 (0.82–7.59) | 1.36 (0.44–4.18) |
| Physical activity | |  |  |
|  | None | Ref | Ref |
|  | 1–2 hours/week | 0.99 (0.36–2.76) | 0.65 (0.23–1.89) |
|  | ≥3 hours/week | 4.08 (0.48–34.64) | 2.95 (0.31–28.55) |
| Body mass index | |  |  |
|  | Normal weight | Ref | Ref |
|  | Overweight | 0.63 (0.22–1.84) | 0.85 (0.27–2.63) |
|  | Obese | 0.26 (0.07–0.90) | 0.35 (0.10–1.18) |
| *Work-related characteristics* | | | |
| Nursing home with units for | |  |  |
|  | Independent residents | 0.89 (0.27–2.90) | 0.59 (0.18–1.93) |
|  | Residents with physical disabilities | 0.70 (0.26–1.87) | 1.77 (0.65–4.80) |
|  | Protected residents | 1.21 (0.36–4.01) | 1.19 (0.34–4.19) |
|  | Residents with addictions | 1.44 (0.14–14.70) | NE |
| Job insecurity | | 1.08 (0.39–3.02) | 0.71 (0.24–2.09) |
| High social support | | 0.64 (0.20–1.98) | 0.33 (0.10–1.04) |
| *Physical and psychological health* | | | |
| Work-related stress | | 1.33 (0.54–3.31) | 1.79 (0.70–4.57) |

Values are relative risk ratios (RRR) with 95% confidence intervals (CI) from a single model including all characteristics listed in the table. Reference outcome category: 0–1 painful sites. For binary characteristics the reference category is absence of the characteristic (for high social support: social support at or below the sample median). Work-related stress: job strain score above the sample median (Job Content Questionnaire). Ref, reference category; NE, not estimable because of sparse data; EU, European Union.
